# A novel framework of multiclass skin lesion recognition from dermoscopic images using deep learning and explainable AI

**DOI:** 10.3389/fonc.2023.1151257

**Published:** 2023-06-06

**Authors:** Naveed Ahmad, Jamal Hussain Shah, Muhammad Attique Khan, Jamel Baili, Ghulam Jillani Ansari, Usman Tariq, Ye Jin Kim, Jae-Hyuk Cha

**Affiliations:** ^1^ Department of Computer Science, COMSATS University Islamabad, Wah Cantt, Pakistan; ^2^ Department of Computer Science, HITEC University, Taxila, Pakistan; ^3^ Department of Informatics, University of Leicester, Leicester, United Kingdom; ^4^ College of Computer Science, King Khalid University, Abha, Saudi Arabia; ^5^ Department of Computer Science, University of Education, Lahore, Pakistan; ^6^ Department of Management Information Systems, CoBA, Prince Sattam Bin Abdulaziz University, Al-Kharj, Saudi Arabia; ^7^ Department of Computer Science, Hanyang University, Seoul, Republic of Korea

**Keywords:** dermoscopic images, skin cancer, deep features, explainable AI, feature selection

## Abstract

Skin cancer is a serious disease that affects people all over the world. Melanoma is an aggressive form of skin cancer, and early detection can significantly reduce human mortality. In the United States, approximately 97,610 new cases of melanoma will be diagnosed in 2023. However, challenges such as lesion irregularities, low-contrast lesions, intraclass color similarity, redundant features, and imbalanced datasets make improved recognition accuracy using computerized techniques extremely difficult. This work presented a new framework for skin lesion recognition using data augmentation, deep learning, and explainable artificial intelligence. In the proposed framework, data augmentation is performed at the initial step to increase the dataset size, and then two pretrained deep learning models are employed. Both models have been fine-tuned and trained using deep transfer learning. Both models (Xception and ShuffleNet) utilize the global average pooling layer for deep feature extraction. The analysis of this step shows that some important information is missing; therefore, we performed the fusion. After the fusion process, the computational time was increased; therefore, we developed an improved Butterfly Optimization Algorithm. Using this algorithm, only the best features are selected and classified using machine learning classifiers. In addition, a GradCAM-based visualization is performed to analyze the important region in the image. Two publicly available datasets—ISIC2018 and HAM10000—have been utilized and obtained improved accuracy of 99.3% and 91.5%, respectively. Comparing the proposed framework accuracy with state-of-the-art methods reveals improved and less computational time.

## 1 Introduction

**Figure 1:**
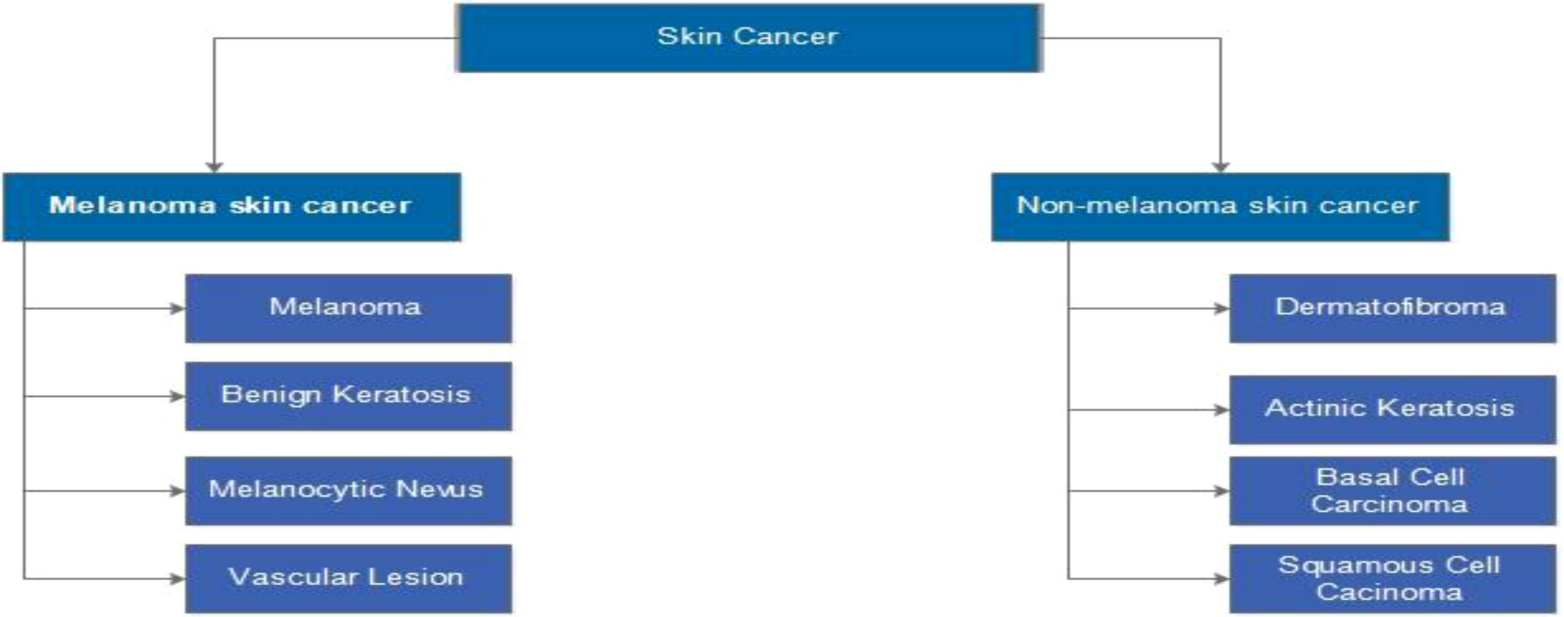
Types of skin cancer such as melanoma or non-melanoma (3).

Many people all over the world have been affected by skin cancer. It occurs when the body’s melanocyte cells grow abnormally and causes damage to the skin’s surrounding tissues (1). The most common types are melanoma, squamous cell carcinoma (SCC), and basal cell carcinoma (BCC). Melanoma can spread quickly from one organ to another (2). Melanoma and non-melanoma are the two most common types of skin cancer, as illustrated in **Figure 1** (3). BCC and SCC are the most common non-melanoma skin cancers (4).

Melanoma has a higher mortality rate than other types of skin cancer. It is the most dangerous type of cancer that must be detected early and is caused primarily by ultraviolet radiation (5). According to the American Cancer Society, there will be over 1 million new cases of melanoma in 2020, with approximately 6,000 deaths (6). Similarly, according to the 19th Skin Cancer Conference, skin melanoma is the most common cancerous growth in both men and women. In 2018, it was discovered that 300,000 new cases were reported (7). The estimated number of deaths in the United States has recently increased compared to reported cases, as shown in **Table 1** for reference (8).

**Table 1:** Comparisons of melanoma cases reported and estimated deaths.

Years	Reported Cases	Estimated Deaths
2020	100350	6850
2021	106110	7180
2022	99780	7650

According to the WHO, sun exposure kills 60,000 people yearly: melanoma killed 48,000 people, and skin cancer killed 12,000 people (9). If melanoma is not detected early, it can spread to the liver, bones, lungs, and brain, making skin cancer patients’ lives difficult and painful (10). As a result, early detection of melanoma is critical for diagnosing skin cancer. As a result, for several decades, the biopsy procedure has been used to examine skin cancers in earlier treatments. It is the most basic approach, and the results are far more consistent. In contrast, the seven-point checklist (11) and the asymmetry, border, color, diameter (ABCD) rule (12) are better than the earlier one. However, these methods require the expertise of a dermatologist to detect cancer. In the last decade, dermatologists have used microscopic and dermoscopy images to diagnose skin cancer (13). Dermatologists examine the images using visual examination. However, dermatologists’ visual inspection and testing of skin lesions take a long time (14). As a result, this procedure necessitates expertise and attention and is time-consuming (15).

As computer vision technology has advanced, the segmentation of medical images has become increasingly important for CAD (16). Dermatologists’ physical screening has become more complex; thus, they use a CAD system to diagnose skin cancer (17). Dermatologists have used these methods to make more timely and effective skin cancer screening decisions (18). CAD methods are much faster and more accurate than human techniques (19, 20). On the other hand, blood vessels, oils, hair, bubbles, and other noise in skin lesion images can make segmentation difficult (21). A CAD system includes several key steps, including contrast enhancement of low-contrast lesions, lesion segmentation, feature extraction, feature selection from the original features, and classification using machine learning (ML) algorithms. Several techniques for contrast enhancement have been developed in the literature, including hybrid filters, color transformations, and haze reduction with a dark channel (22). This step’s improved performance improved the accuracy of lesion segmentation, which has an impact on useful feature extraction (23). Several lesion segmentation techniques, such as thresholding, saliency and region growing, and clustering, have been developed in the literature. In traditional techniques, the segmented images are used for feature extraction; however, the problem of irrelevant features is solved by computer vision researchers using feature selection techniques (24). Using feature selection techniques, the best features are chosen from the original extracted features, resulting in a reduction in computational time (25, 26).

**Figure 2:**
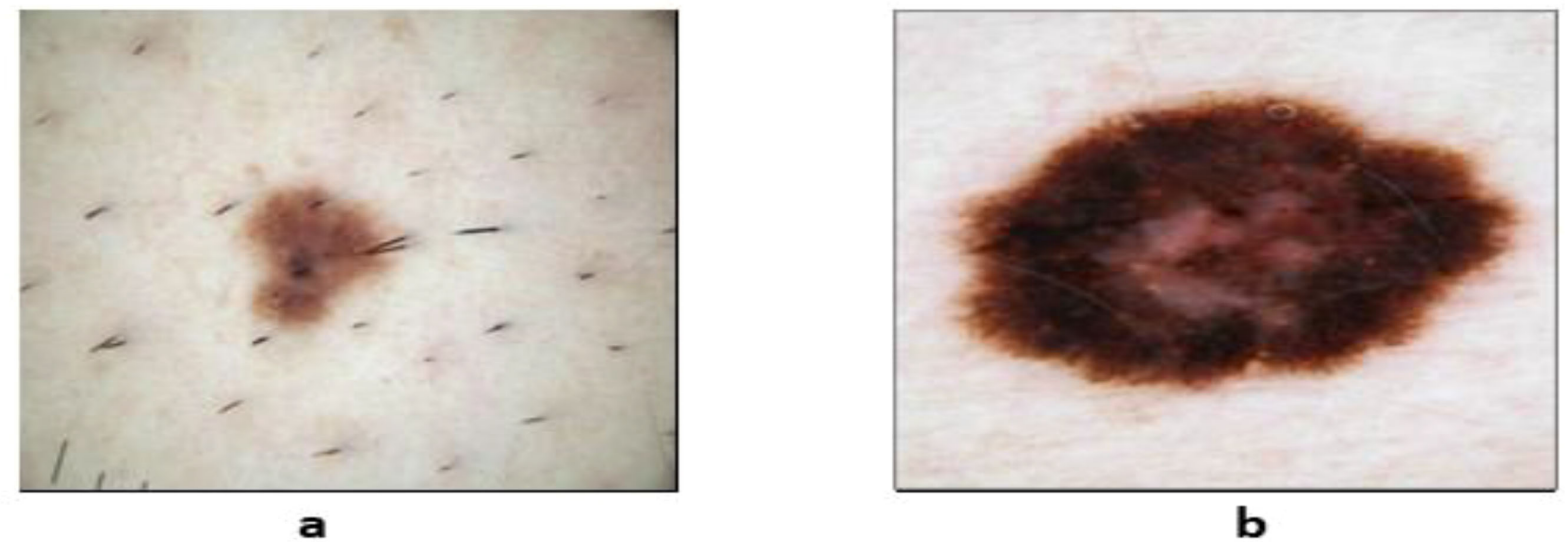
Sample dermoscopic images: **(A)** benign skin lesion and **(B)** malignant skin lesions (29).

As a result, to perform effectively in skin lesion segmentation tasks, deep learning (DL) algorithms play an important role in achieving a high accuracy level (27). Convolutional neural networks (CNNs) are commonly used in medical image processing for melanoma detection. Researchers have suggested several deep learning–based models. Several methods produce impressive results when it comes to skin lesion segmentation (28). Fully CNNs (FCNs), CNNs (CNNs), deep CNNs (DCNNs), fully convolutional residual networks (FCRNs), and U-Net are some of the techniques that can be used to segment skin lesions. Skin lesion images can be classified to aid in detecting skin cancer. Images of skin lesions could be used to detect any type of skin cancer. **Figure 2** depicts the distinction between benign and malignant tumors.

Supervised classification is used in computer vision to map data into various classes and categories. A labeled dataset is required. Traditional classification approaches such as decision tree (DT), artificial neural network (ANN), support vector machine (SVM), and a variety of additional classifiers have been implemented to classify skin lesion images (30). DL methods have recently produced cutting-edge results in skin lesion analysis to the point where images are now being used to diagnose skin diseases (31).

### 1.1 Problem statement

Since the last few years, a significant amount of research has been conducted in this domain using deep learning methods. Despite this, numerous challenges exist in this domain, such as the contrast problem of infected lesions, variation in the shape of lesions, and color similarity of different skin lesion classes. Based on these challenges, there is room for improvement in lesion detection and multiclass classification accuracy. The proposed research addresses the following issues: low-contrast and noisy skin lesions are extracted as the incorrect region of interest, lowering the final step’s accuracy. The change in shape, texture, and color of skin lesions from different skin classes increases the accuracy of misclassification. Unbalanced datasets train the incorrect model and provide higher accuracy for the higher weight class. The similar appearance of skin lesion types, such as benign and akiec, creates a high risk of misclassification, and feature extraction from a single source is insufficient for improving the accuracy of multiclass problems.

### 1.2 Major contributions and manuscript organization

The major contributions of this work are as follows:

- Performed a data augmentation based on modified mathematical formulations, fine-tuned two pretrained deep models, and extracted features from the global average pooling layer.
- The extracted features were serially fused and selected using Improved BAT Optimization Algorithm (IBOA), a new and improved optimization algorithm. A cost function based on minimization is developed that can select the best features. In addition, an entropy was added to handle uncertainty.
- For the visualization of important parts of the image, an explainable artificial intelligence (AI) approach, such as GradCAM, is used.
- An ablation study was conducted, and the results were compared to recent techniques.

The manuscript is organized where Section 2 describes the related work based on skin lesion classification. Section 3 describes the proposed methodology, followed by Section 4, which elaborates and discusses the datasets, experimental setup, results, and comparisons with existing methods. Finally, the conclusion is given in Section 5.

## 2 Related work

Image segmentation (32) separates the infected skin (33) from healthy skin, effectively determining skin diseases. The Res-Unet approach combines the U-Net and the Res-Net designs for dynamically segmenting lesion borders. The author employed an algorithm based on morphological operations for hair removal, substantially improving the segmentation performance (34). Razmjooy et al. (35) used the Quantum Invasive Weed Optimization Technique to create an optimal neural network (NN) for separating the skin lesion region. Sreelatha et al. (36) published a melanoma segmentation approach that is dependent on the Gradient and Feature Adaptive Contour model for diagnosing the earliest stage of melanoma with 98.64% accuracy. To reduce noise and speed up execution, the suggested image segmentation approach employed preprocessing and noise-elimination methods. Yacin Sikkandar et al. (37) employed the inception model for feature extraction and provided a grab-cut segmentation technique to segment the preprocessed images.

The ability to extract features and train based on automated feedback is the most significant advantage of CNN-based techniques. Deep learning models such as VGG, AlexNet, ResNet, and Xception have made significant progress in recent years (38). Because of their effectiveness, these models have also been used in CAD systems in various research studies. Saba et al. (39) have provided a technique for automatically diagnosing skin lesions by utilizing a DCNN in their work. Their method consists of three primary processes, which are the improvement of contrast, the extraction of lesion boundaries using the CNN, and the collection of features using the Inception V3 model. The accuracy of the experiments on the ISBI-2016, ISBI-2017, and PH2 datasets was 95.1%, 94.8%, and 98.4%, respectively. Utilizing the HAM10000 dataset, the authors (40) built a classification model using 10 distinct pretrained CNNs and SVMs to extract the features. The model achieved an accuracy of 90.34%. In another paper, the researcher (41) presented the Nasnet-large deep model feature extraction utilizing transfer learning (TL). The experiment is conducted using HAM10000 and ISIC2018, both available to the public. The accuracy obtained on both datasets is 93.40% and 94.36%, respectively. In this (42) study, the researcher evaluated an effective automated system for skin cancer classification. An image recognition model called MobileNet was employed in this study. It was developed using over 1.2 million images taken as part of a 2014 ImageNet Challenge. The TL technique is used to fine-tune it on the HAM10000 dataset. The model attained 83.1% average accuracy for seven classes in this dataset. The study (43) used a DCNN model and a DL technique to classify skin lesions correctly. To calculate the performance of this DCNN model, certain pretrained models were employed, such as VGG-16, MobileNet, DenseNet, AlexNet, and ResNet, which were used for TL. However, when applied to the HAM10000 dataset, this model achieved accuracy rates of 93.16% during training and 91.93% during testing. Villa-Pulgarin et al. (44) developed the DenseNet-201, Inception-V3, and Inception-ResNet-V2 deep models using TL. The dataset HAM10000 was used to evaluate these models in which the DenseNet-201 model performed best on the International Skin Imaging Collaboration (ISIC) 2019 dataset with an achieved accuracy of 93%.

The researcher presented the inceptionV3 model in (45), which used ISIC 2019 and 2020 datasets and achieved an accuracy of 86.90%. Similarly, in (46), researchers developed an effective deep-learning strategy for classifying various types of skin lesion images. First, the author used data augmentation to improve the skin cancer dataset. The image features of skin lesions are then extracted using a fine-tuned pretrained deep learning model called Xception. Next, TL is used by freezing the first 36 layers of the model and retraining the remaining 35 layers of the previously trained model. Finally, the pretrained model’s final layer was deleted and it was replaced with a dense layer. This allows us to categorize eight different types of skin lesions. The system model was evaluated with 95.96% accuracy using the ISIC 2019 dataset in this paper.

## 3 Proposed methodology

**Figure 3:**
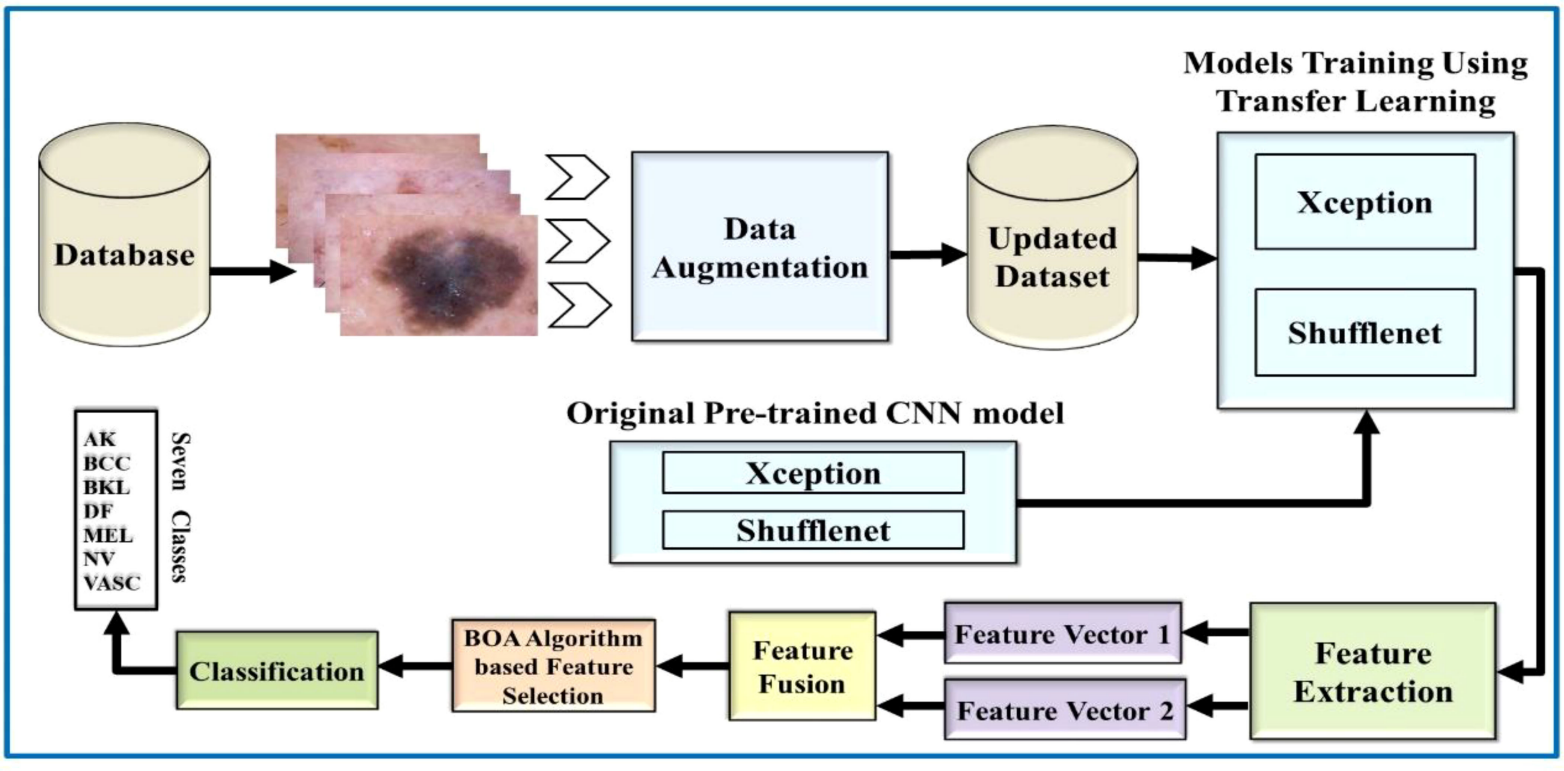
Flow diagram of the proposed skin lesion classification using two-stream deep learning architecture.

This work is based on the skin lesion classification method to solve the existing issues and address the aforementioned challenging problems. Based on the existing literature review, there is still much room to improve accuracy. Therefore, **Figure 3** illustrates the major steps of this challenging research work. According to **Figure 3**, the proposed methodology made use of two publicly available datasets, HAM10000 and ISIC 2018. Data augmentation is used in these datasets to increase the training data. Then, TL is used to train pretrained models such as Xception and ShuffleNet. The global average polling layer extracts features from both deep models, which are fused using the Serial-Threshold fusion approach. The fused feature vector is then subjected to the BOA feature selection/optimization method to obtain the optimal feature vector. Finally, the optimal feature vector is classified using various ML algorithms such as SVM and NNs.

### 3.1 Data augmentation

A large, comprehensive dataset will help a machine learning model perform better and more accurately. This is due to the large data required to train a model. As a result, data augmentation has recently become increasingly important in deep learning to achieve good performance.

This procedure consists of a 90-degree rotation, a right-to-left flip (RLF), and an up and down Flip (UDF). These steps are repeated several times until each class has 6,000 images. As a result, the updated datasets contain 36,000 images, a significant increase over the original number of images, which was 10,000 for each dataset. The mathematical procedures are performed in the following ways.

*a)* Consider the image datasets *a* = {*x*_1_,*x*_2_, … …, *x_n_*} (40), where *x_n_* ϵ *UF* is a representative image from the dataset. Let *x_n_* have a total of *D_k_* pixels; the homogeneous pixel matrix coordinates *D_k_* or *X_n_* are as follows:


(1) $$D_{k}=\left[\begin{array}{ccc} P_{1} & Q_{1} & 1\\ P_{2} & Q_{2} & 1\\ \vdots & \vdots & 1\\ P_{n} & Q_{n} & 1 \end{array}\right]$$

*b)* An input image with a size of 256 × 256 × 3, which is denoted as *UF_a,b,c_* where a, b, and c denotes rows, columns, and channels, respectively, to compute *UF_a,b_* ϵ R^a×b^. The flip-up (UDF) procedure is computed in the following manner (41):

*Firstly, UF^t^* is the transpose of the original image and is taken out using the equation given below:


(2) $$UF^{t}=UF_{b,c}$$

*Secondly*, *UF^V^* represents the vertical image. The vertical flip procedure is described in the following equation:


(3) $$UF^{V}=UF_{(m+1-a)b}$$

*Thirdly*, *UF^H^* represents the horizontal image. The horizontal flip procedure is described in the following equation:


(4) $$UF^{H}=UF_{a(n+1-b)}$$

*Finally*, the images from the selected datasets are rotated to 90° using the following formulation:


(5) $$Rotate=\left[\begin{array}{ccc} COS\;\alpha & -Sin\;\alpha & 0\\ Sin\;\alpha & COS\;\alpha & 1\\ 0 & 0 & 1 \end{array}\right]$$

The whole augmentation process is combined and shown in **Equation 6**.


(6) $$Data\;Aug=\left(UF^{V},UF^{H},Rotate\right)$$

### 3.2 Skin cancer model training

**Figure 4:**
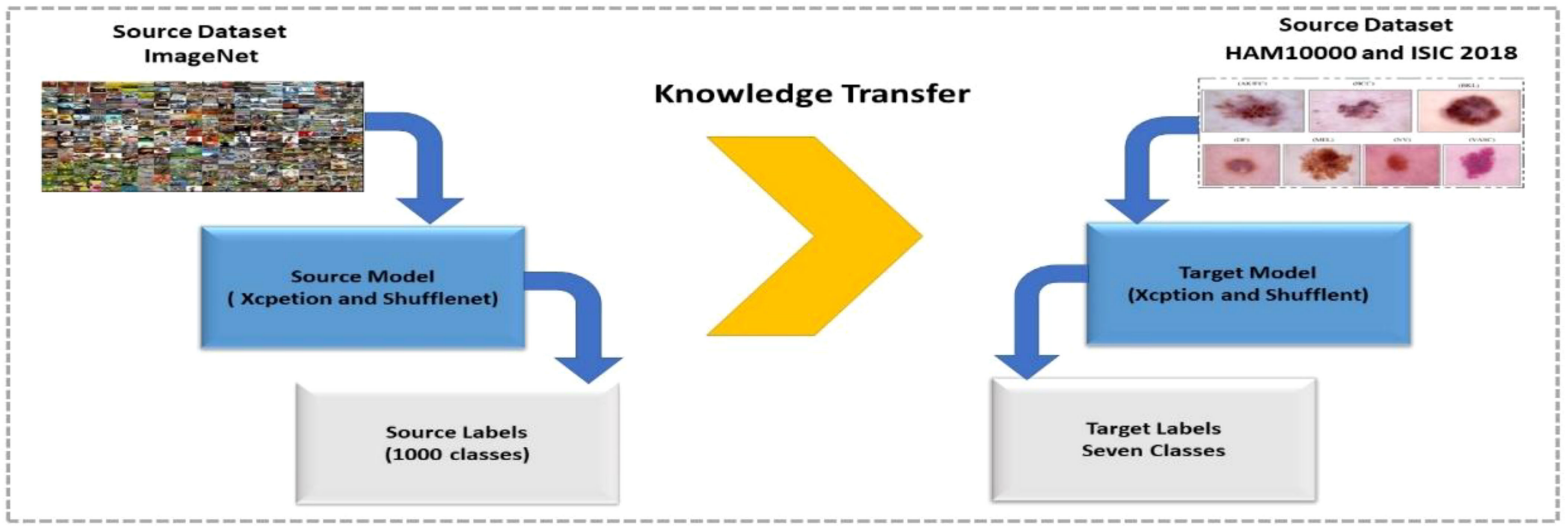
Training of fine-tuned models using transfer learning for skin lesion classification.

TL is a type of machine learning in which a model is trained for one task and then used as the foundation for a different task. TL can be effective when one domain’s dataset is significantly smaller than the associated domain (47). **Figure 4** depicts a graphical representation of how TL can be used to solve any problem. The pretrained model, which included Xception and ShuffleNet, was trained on ImageNet before being fine-tuned and trained on the target datasets for this work.

Suppose that *S_d_* is a source domain and *S_t_* is a source task, and then, they are defined as {(*S_d_*,*S_t_*)|*p* = 1,2,3,4, …., *n^s^*}. The notation indicates the target domain *T_d_* while the notation *T_s_* indicates the target source; the whole structure is then expressed as {(*T_d_*,*T_t_*)|*q* = 1,2,3,4, …., *n^t^*}. TL uses the following objective function to determine how much information from the source domain should be transferred into the target domain.


(7) $$F^{tq}\left(q=1,2,3,4,\ldots ..,n^{t}\right)$$

### 3.3 Deep feature extraction

The major step is extracting features using deep models, including Xception and ShuffleNet, after performing data augmentation and transfer learning on pretrained models. Several recent studies have provided effective solutions and results considering numerous challenges in medical imaging when deep feature extraction is adopted for classification. Regarding the current problem, various skin lesion image elements, including color, shape, angle, geometry, and object dimensions, are the focus of interest.

Furthermore, the deep features, in contrast to handcrafted features that provide low-level features, are more effective and provide highly discriminative and superior outcomes. This could be achieved by employing a standard CNN model consisting of multiple convolutions, pooling, normalization layers, an FC layer, and finally, a classification layer (48). Thus, we use deep models named Xcpetion and ShufflNet to extract the features.

#### 3.3.1 Fine tuning Xception model

**Figure 5:**
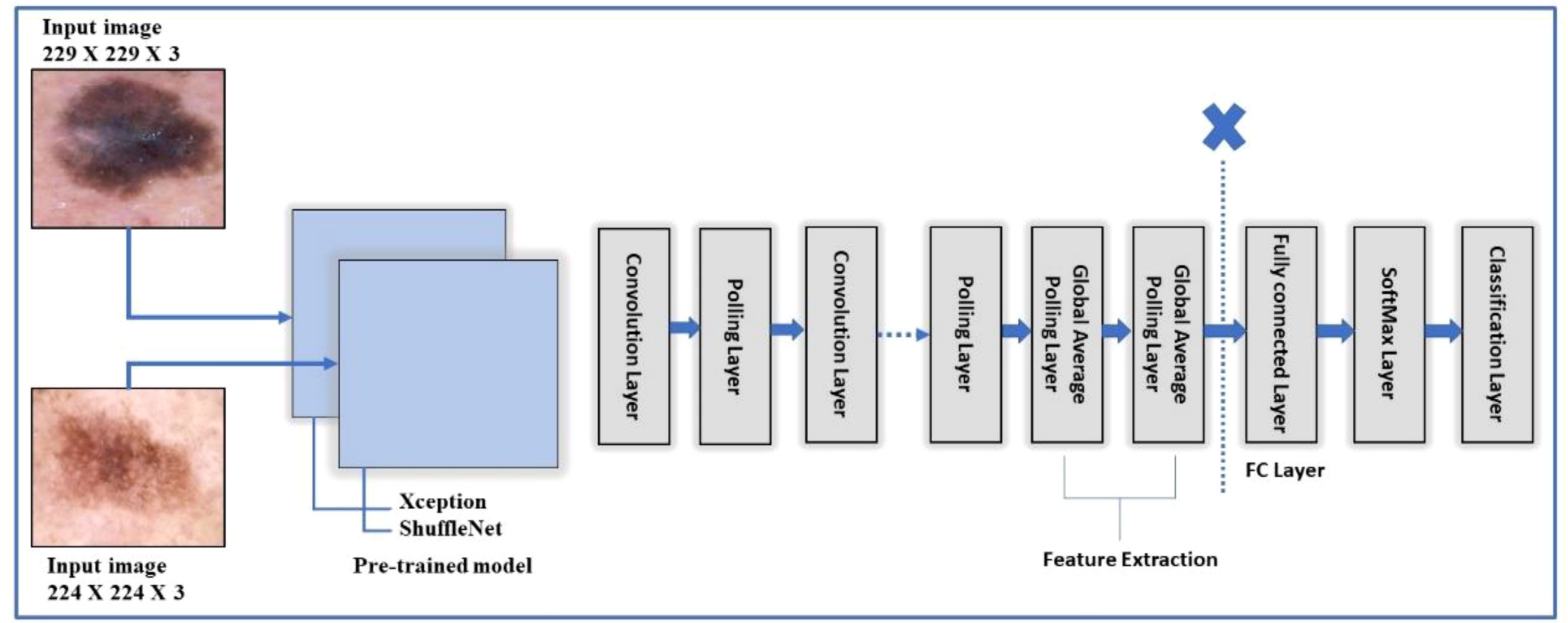
A framework of Xception and Shufflenet deep model for feature extraction of the proposed skin lesion classification.

The model organizes the convolution layers in a novel way and separates them depth-wise. The key feature of Xception is its extreme inception architecture. The feature extraction property of the network is formed using 36 convolutional layers. Among all, except for the first and last modules, the 36 convolutional layers are organized into 14 modules, each of which is surrounded by linear residual connections (49). In the fine-tuning process, the final three layers are deleted, and a new fully connected (FC) prediction layer is added, connected to two further layers: new_softmax and new_classoutput, respectively. After that, transfer learning is utilized, and the whole fine-tuned setup is trained on the selected skin datasets. Target datasets were split into 50:50 instead of 70:30 training: testing. This indicates that 50% of the images in each class were used for training, while the remaining 50% were used for testing. The hyperparameters for this network are: total number of epochs is 3, the learning rate is set to 0.0001, and the batch size is 8. In addition, the learning technique rmsprop optimizer is chosen, and the mini-batch size is set at 16. After training, features are extracted from a layer which is referred to as “avg1,” and from the global average pool layer instead of the FC layer. This way, the feature vector size could be (× 2048) features, where N represents the number of training samples. The obtained feature vector from this model is named FV1. **Figure 5** shows the fine-tuning process of the Xception model.

#### 3.3.2 Fine tuning ShuffleNet model

ShuffleNet is a very efficient architecture based on the Xcpetion and ResNeXt. ShuffleNet outperforms the state-of-the-art architecture MobileNet (50) by a large margin, with an absolute 7.8% reduced ImageNet top-1 error at 40 MFLOPs. In the fine-tuning process, same as the Xcpetion model, the final three layers are deleted, and a new fully connected (FC) layer named new_node_202 is introduced, which is connected to two further layers that are softmax (new_node_203) and classification layer (new_classificationLayer_node) respectively. The fine-tuned architecture is trained on the selected datasets following the split ratio, as mentioned in section 4.3.1. After training, features are extracted from a layer referred to as “node_200,” the global average pool layer instead of the FC layer. This way, the feature vector size could be N × 544 features, where N represents the number of training samples. The obtained feature vector from this model is called *FV*2 **Figure 5** shows the ShuffleNet and Xception models collectively to depict the fine tune process.

### 3.4 Feature fusion

Let us say that *X* and *Y* are two different feature spaces defined on the pattern sample space that is δ. The two feature vectors for an arbitrary sample *p* ϵ δ are *σ* ϵ *Y* and *τ* ϵ *Y*. $u={(}_{\tau }^{\sigma })$ defines the serial combination features of *p*. If the feature vector has *σ = k* dimensions and the feature vector has *τ = n* dimensions, then the serial combined feature will have (*k* + *n*) dimensions. A (*k* + *n*)-dimensional serial combined feature space is formed by all obtained feature vectors from the selected set of patterns. Then, concatenation is done as follows:


(7) Feature fusion=(FV1,FV2)

As a result of **Equation 7**, the size of obtained feature vector is (*N* × 2,592) features, where *N* represents the training images. However, the feature vector size is too large to classify the skin lesion images and requires a huge computation time. Therefore, a BOA optimizer is employed to obtain an optimal feature vector for accurate and robust classification in the minimum time to minimize the size of the fused feature set.

### 3.5 Feature selection using Butterfly Optimization Algorithm

In this work, an algorithm for optimum feature selection called the Butterfly Optimization Algorithm (BOA) is employed to enhance the classification accuracy in the later step.

The algorithm works on butterflies’ behavior as they search for food served. This notion serves as an inspiration for a new nature-based meta-heuristic algorithm called the BOA (51). The following are the characteristics of butterflies that are the basis of BOA:

- It is generally accepted that all species of butterflies produce a smell that serves to other species of butterflies.
- Each butterfly flits around at random or in the direction of the best butterfly, the butterfly that gives off the strongest fragrance.
- The objective function map determines butterfly stimulus intensity.

The perceived magnitude of the fragrance (F) is specifically described in the BOA as a function of the stimulus’ physical intensity:


(8) $$F=s\;K^{x}$$

where S ϵ [0,∞] denotes the sensory modality; K is the stimulus intensity s linked to the encoded objective function; and *x* ϵ [0,1] denotes the power exponent based on modality, which denotes the changing degree of fragrance absorption.

The two most important stages of the BOA are the global search and the local search. The former can encourage the butterflies to migrate in the direction of the best butterfly, which might be symbolized as


(9) $$b_{j}^{t+1}=b_{j}^{t}+\left(d^{2}\times c^{*}-b_{j}^{t}\right)\times F_{j}$$

where $b_{j}^{t}$ is the location of the jth butterfly at time *t* in the iteration. In Equation 10, the symbol *c* stands for the position that is now in best place. *F_j_* is denoted by for fragrance that the jth butterfly produces, and d is a random number in the range [0, 1].

The second approach is accomplished using a local search, which may be symbolized as


(10) $$b_{j}^{t+1}=b_{j}^{t}+\left(d^{2}\times b_{j}^{t}-b_{m}^{t}\right)\times F_{j}$$

where $b_{j}^{t}$ and $b_{m}^{t}$ are the *i*th and *m*th butterfly locations in the solution space, respectively.

In addition, the BOA makes use of a switch probability, denoted by the symbol *p*, to switch between a standard global search and an intense local search.

Given the information presented above, the pseudo-code of original BOA is represented in Algorithm 1.

Algorithm 1 **BOA-based feature section algorithm.**

**Begin**
Makes objective function *f*(*b*), where *b* = (*b*
_1_,*b*
_2_, …., *b_Dim_
*), and *Dim* is the dimension.
Create a starting population P with N butterflies, *P_i_
*(*i*=1,2, …., *N*)
Stimulus intensity *k_i_
* at is *p* defined by the *f*(*P*)*
_i_
*
Determine the sensor modalities *S*, and *x* is the power exponent and switch probability *p*.
**While** condition don’t false **do**
**for each** in the population P of butterfly bf **do**
Applying Equation (8), determine fragrance F
**end for**
Find the best butterfly bf
**for each** in the population P of butterfly bf **do**
Create a random number *d* range from 0 to 1
**If (*d***< ***p***)
Equation (9) is used to do global search for best butterfly
**else**
Equation (10) is used to do local search for randomly chose butterfly
**end if**
**end for**
The value of the power exponent *x* is updated
**end while**
Provide the finest solution and the greatest value
**End Begin**

In this proposed work, the number of butterflies for the optimal solution is 10, and the maximum iteration is 100 to obtain the best feature vector. After applying this BOA, the feature vector can reduce the features that are (× 943) for the HAM10000 dataset and (*N* × 1,080) for the ISIC 2018 dataset, where *N* represents the number of training images. **Table 2** reflects a significant reduction in the feature vectors obtained in Section 4.4 before and after applying BOA.

**Table 2:** Comparison of features before and after BOA.

Datasets	Before apply BOA	After apply BOA
HAM10000	*N* × 2592	*N* × 943
ISIC 2018	*N* × 2592	*N* × 1080

### 3.6 Classification

An optimal feature vector from Section 4.5 is employed to perform classification. This proposed work evaluates supervised classification results using 10 classifiers, including Narrow Neural Network, Wide Neural Network, Bilayered Neural Network, Trilayered Neural Network, Quadratic SVM, Cubic SVM, Fine KNN, Ensemble Bagged Tree, and Fine Tree.

The skin dataset is separated into ten equal partitions for all the experiments, with a cross-validation size of 10-folds. The first fold is used as a testing set when the experimental model is started, while the rest are utilized for training the model. Similarly, until the model is finished, all 10 folds repeatedly operate as training and testing sets. All the classifiers are used on data that are divided into folds. The classifiers learn the fold criteria and then predict the outcome based on the testing partition.

## 4 Results and discussion

The entire experimental analysis results are presented in this section based on the proposed methodology. Because the primary goal of this research is to improve accuracy while reducing computational costs. As a result, the proposed method is tested using two different sets of data: HAM10000 and ISIC2018. These datasets are freely available to the public. A total of 10 different classifiers are used for experimentation, including Neural Network, Quadratic SVM, Fine Tree, Fine KNN, and Ensemble Bagged Tree. The evaluation metrics of these classifiers are discussed, including sensitivity, F1-Score, precision rate, accuracy, FPR, and testing time. All experiments are written in MATLAB 2021a and run on an Intel Core i7 7th generation CPU with 8GB of RAM and a 1TB hard drive.

### 4.1 HAM10000 dataset

**Figure 6:**
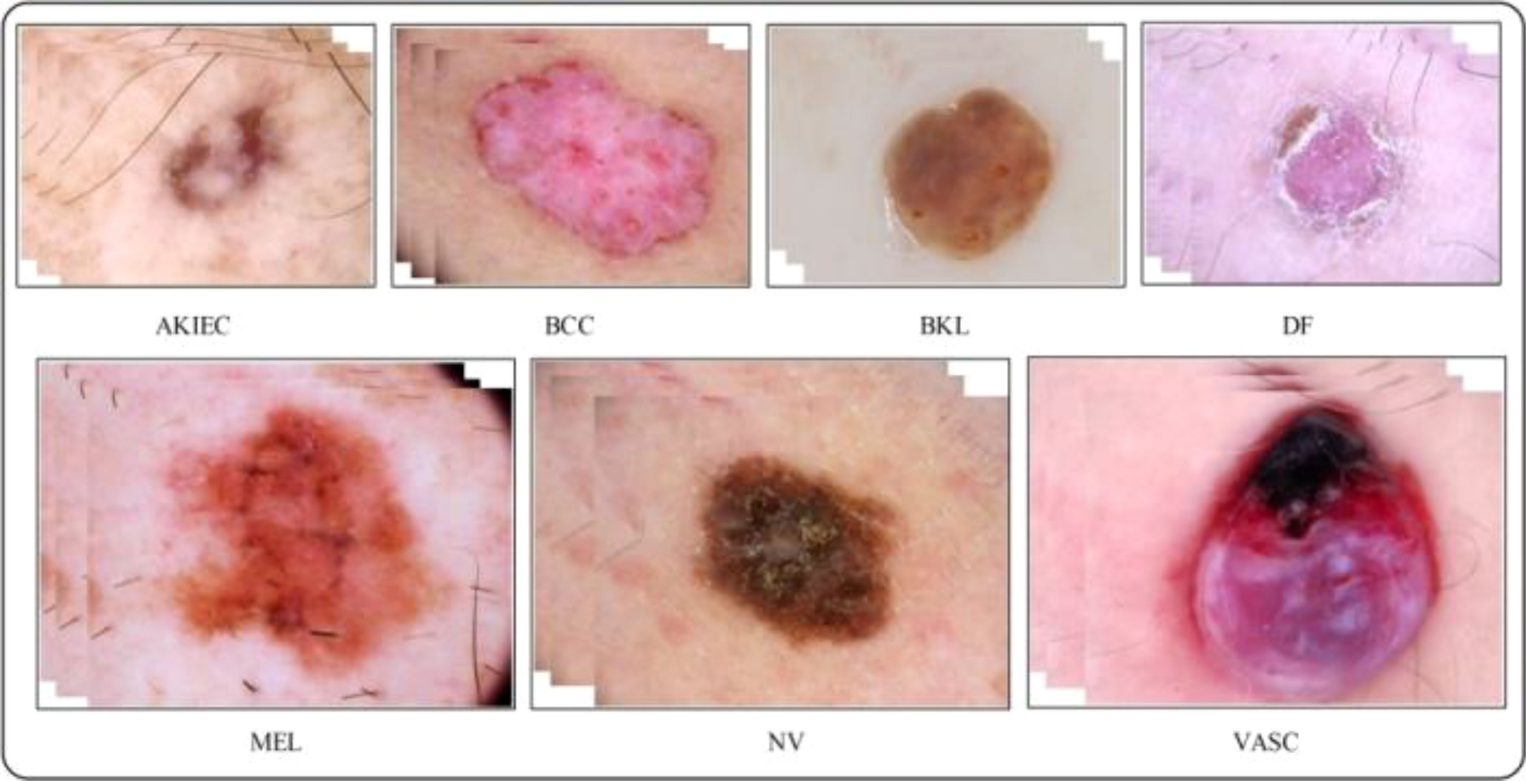
Sample images of the HAM-10000 datasets (52).

A total of 10k dermoscopy images are included in the HAM10000 dataset “Human Against Machine with 10,000 Training images,” the most significant datasets accessed through the ISIC repository (52). The dataset is a collection produced included 1,113 images of melanomas (mel), 327 images of AK (actinic keratosis), 514 images of basal cell carcinomas (bcc), 1,099 images of benign keratoses (bkl), 115 images of dermatofibromas (df), 6,705 images of melanocytic nevi (nv), and 142 images of vascular (vasc) skin lesions (53). The skin lesion images in the dataset are split between men and women, with men being 54% and women being 45% of the images. Classifying these skin classes is not easy because the dataset is complex, containing multiple skin lesion images, and there are issues concerning low interclass variation and high intraclass variation. As a result, there is a strong possibility that a high misclassification rate will occur. **Figure 6** displays a few examples of the images available.

### 4.2 ISIC 2018 dataset

**Figure 7:**
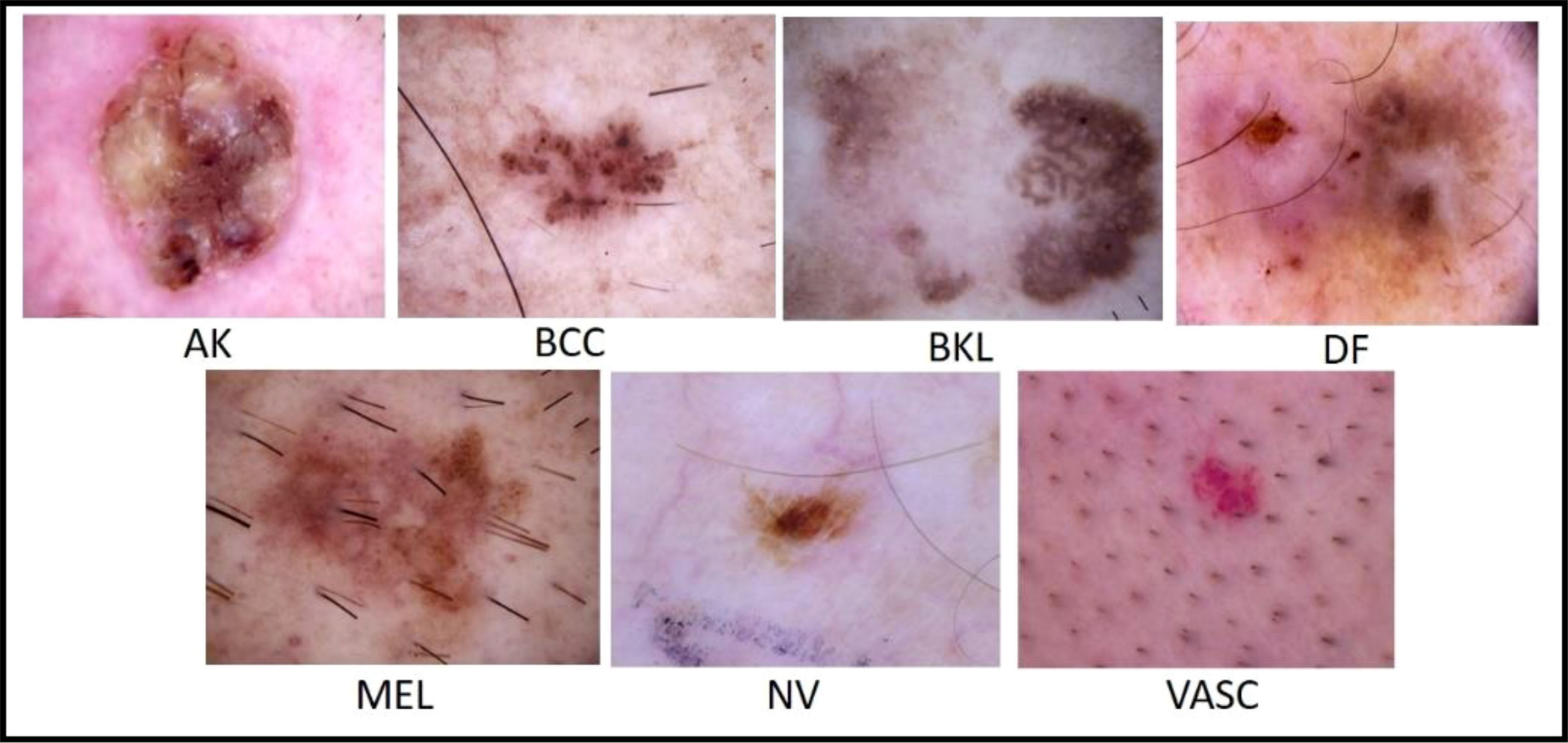
Sample images of the ISIC 2018 dataset (56).

The ISIC has published a large-scale dataset of dermoscopy images called the ISIC 2018 dataset. This dataset comprised more than 12,500 images. The dataset performs three tasks: lesion segmentation, attribute identification, and disease classification (54). This dataset contains almost 10,000 images of seven different types of classes for the classification task (55). **Figure 7** shows a few samples of ISIC 2018 dataset images. The ISIC 2018 challenge includes two major issues: first, certain classes have a restricted number of images, and second, the imbalanced number of images in different classes makes it difficult for the classifier to correct classification.

### 4.3 Experiments

Four separate experiments are used to compute the proposed framework’s results on both datasets:

- Fine-tuned Xception deep model features
- Fine-tuned Shufflenet deep model features
- Feature Fusion of both deep models
- BOA-based feature selection

### 4.4 HAM10000 dataset results

**Table 3** displays the classification results on the HAM10000 using Xcpetion deep features. **Table 3** shows that Cubic SVM performed admirably, with an accuracy level of 99.1%. While it also outperforms its competitors on other metrics, its recall rate is 99.18%, its precision rate is 99.22%, its F1-Score is 99.18, and its FNR is 0.82%. Furthermore, the Cubic SVM classifier has the longest computation time during the training phase at 1,017.9 seconds (sec). Fine Tree classifiers, on the other hand, have the shortest computation time of 100 seconds.

**Table 3:** Results of classification incorporating Xception deep features applied on the HAM10000 dataset.

Classifiers	Accuracy (%)	Recall (%)	Precision (%)	F1 Score	FPR	Time (sec)
Narrow NN	98.9	98.97	98.88	98.17	1.03	469.27
Medium NN	98.9	99.01	99.02	99.01	0.99	261.74
Wide NN	98.9	99.02	99.05	99.03	0.98	334.87
Bilayered NN	98.8	98.92	98.92	98.92	1.08	500.02
Quadratic SVM	99.0	99.1	99.17	99.12	0.88	1009.5
**Cubic SVM**	**99.1**	**99.18**	**99.22**	**99.18**	**0.82**	**1017.9**
Coarse Gaussian SVM	98.7	98.77	98.44	99	1.23	1931
Fine KNN	99.1	99.15	99.2	99.16	0.85	2002
Ensemble Bagged Tree	98.8	98.9	98.9	98.9	1.1	327.58
Fine Tree	94.9	95.15	95.15	95.18	4.85	100.25

Bold show the significant value.

**Table 4** displays the classification results on HAM10000 using ShuffleNet deep features. **Table 4** clearly shows that the Fine KNN achieved the highest accuracy of 98.9%. It also outperforms its competitors on other metrics, such as the recall rate 99.15%, precision rate 99%, F1-Score 99.06, and FNR 0.85%. Furthermore, due to the complexity of the dataset, the compute time of the Fine KNN classifier during the training phase is 920.22 seconds (sec). However, the classifier Medium NN takes the least time (116.01 seconds). The Fine Tree classifier, on the other hand, has a shorter computation time but performs poorly in comparison to its competitors. As a result, we consider the Medium NN classifier because it is more accurate than the Fine Tree.

**Table 4:** Results of classification incorporating Shufflenet deep features applied on the HAM10000 dataset.

Classifiers	Accuracy (%)	Recall (%)	Precision (%)	F1 Score	FNR	Time (sec)
Narrow NN	96.5	96.82	96.75	96.68	3.18	197.3
Medium NN	97.3	97.55	97.51	97.52	2.45	116.01
Wide NN	97.6	97.78	97.72	97.74	2.22	130.81
Bilayered NN	96.6	96.84	96.81	96.82	3.16	284.3
Quadratic SVM	98.1	98.3	98.34	98.3	1.7	546.39
Cubic SVM	98.5	98.62	98.64	98.62	1.38	585.07
Coarse Gaussian SVM	96.5	96.71	96.77	96.72	3.29	986.83
**Fine KNN**	**98.9**	**99.15**	**99**	**99.06**	**0.85**	**920.22**
Ensemble Bagged Tree	95.0	95.13	95.34	95.32	4.87	209.95
Fine Tree	69.3	68.62	71.21	69.92	31.38	63.97

Bold show the significant value.

**Table 5** shows the classification results on HAM10000 using deep combinatorial features extracted from the Xception and ShuffleNet architectures. For testing purposes, the deep features are serially fused. Quadratic SVM achieved the highest accuracy level of 99.3% in this test. Compared to its competitors, it also performs well on other metrics such as the recall rate 99.32%, precision rate 99.37%, F1-Score 99.34, and FNR 0.68%. Furthermore, the Quadratic SVM classifier’s computation time during the training phase is 1,305.5 seconds (sec), which is higher and ranks second. However, the Fine Tree classifier has the shortest computation time of 139.4 seconds.

**Table 5:** Results of classification incorporating feature fusion of both deep models on the HAM10000 dataset.

Classifiers	Accuracy (%)	Recall (%)	Precision (%)	F1 Score	FNR	Time (sec)
Narrow NN	99.1	99.17	99.15	99.14	0.83	937.84
Medium NN	99.1	99.18	99.17	99.16	0.82	336.21
Wide NN	99.1	99.15	99.2	99.16	0.85	1,787.2
Bilayered NN	99.0	99.17	99.17	99.17	0.8	380.38
**Quadratic SVM**	**99.3**	**99.32**	**99.37**	**99.34**	**0.68**	**1,305.5**
Cubic SVM	99.3	99.34	99.4	99.36	0.66	1,391.2
Coarse Gaussian SVM	98.9	98.97	99.04	99	1.03	2,676
Fine KNN	99.2	99.31	99.32	99.3	0.69	2,471.7
Ensemble Bagged Tree	98.8	98.87	98.87	98.87	1.13	398.88
Fine Tree	94.9	95.11	95.2	95.14	4.89	139.4

Bold show the significant value.

**Figure 8:**
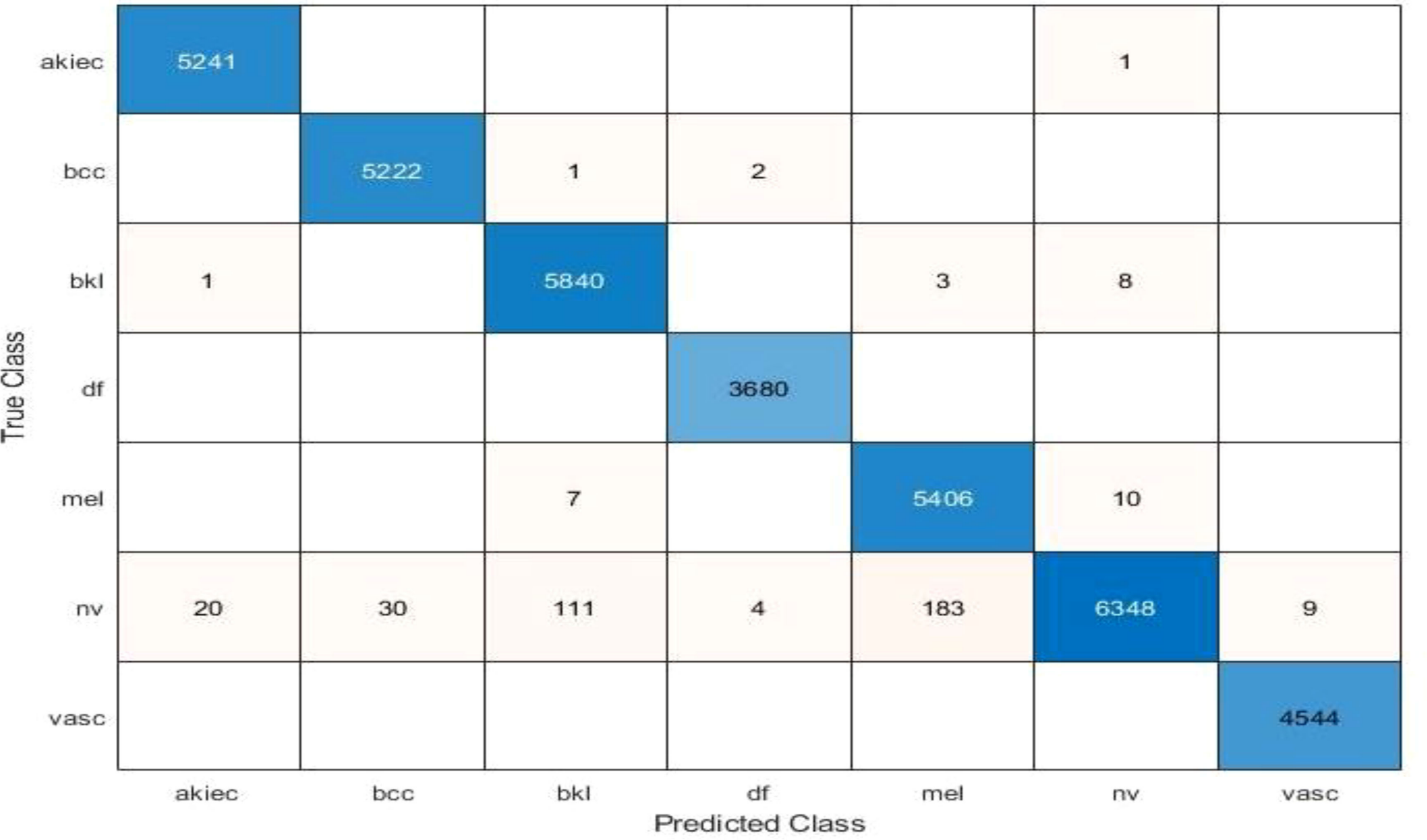
Confusion matrix of recall on Fine KNN using BOA feature selection.

**Figure 9:**
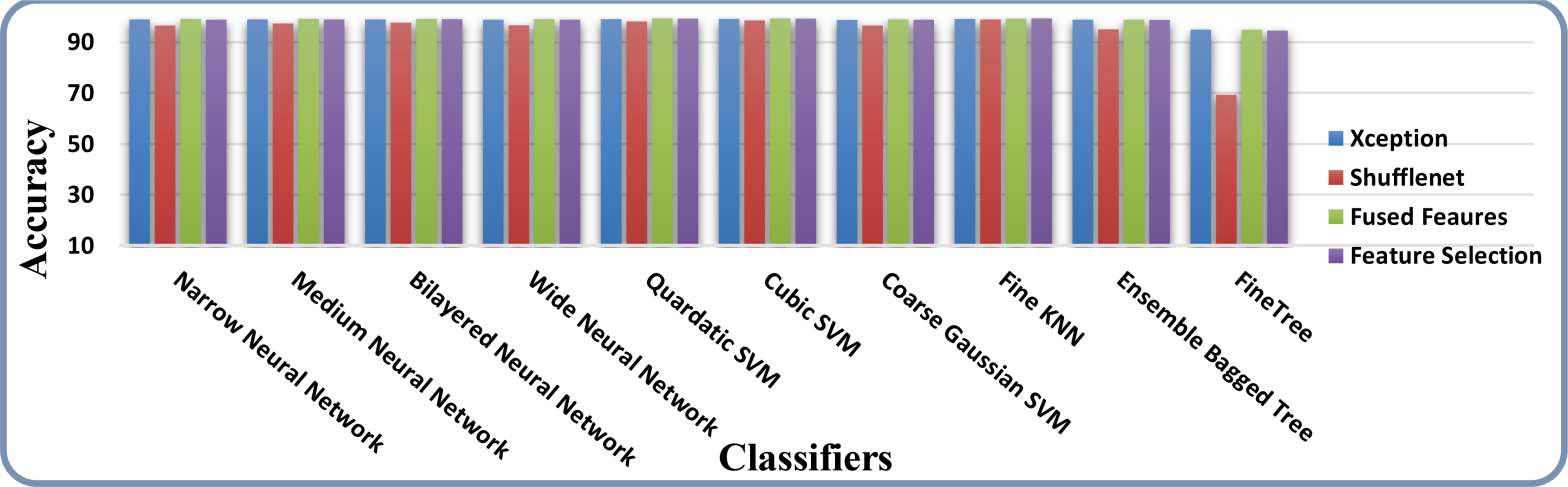
Accuracy comparison of all the intermediate steps on the HAM10000 dataset.

**Figure 10:**
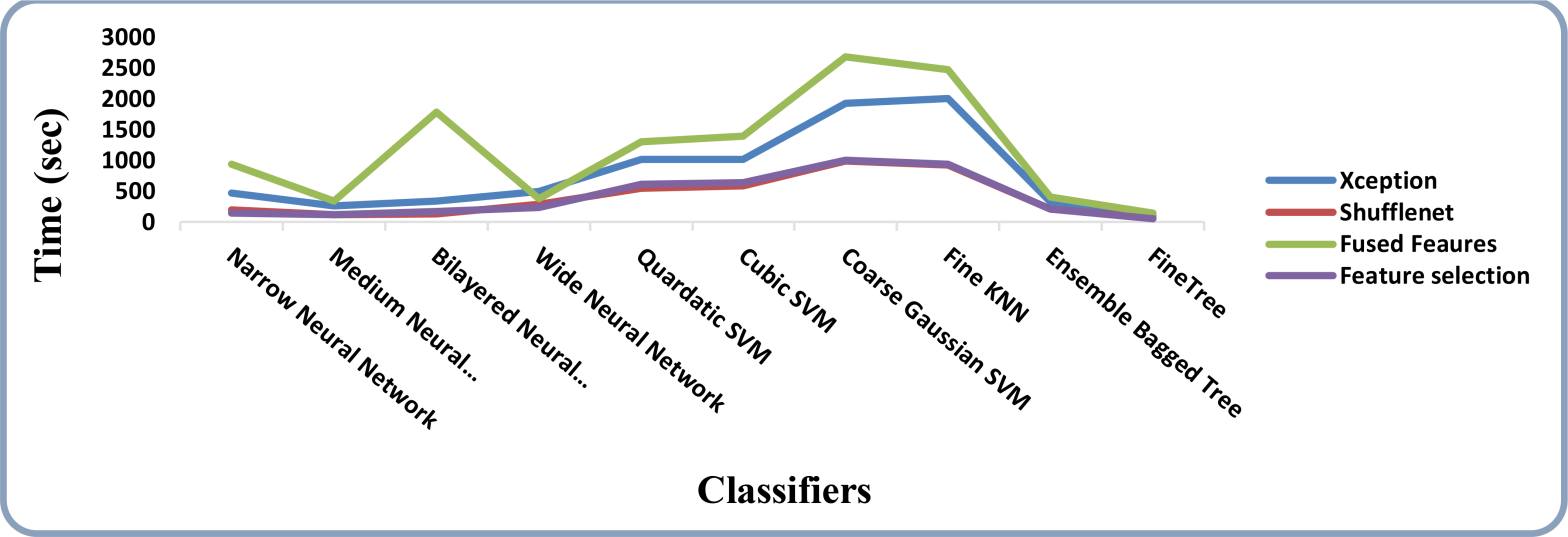
Time-based comparison of all the intermediate steps on the HAM10000 dataset.

The results of feature selection using the optimization algorithm BOA are shown in **Table 6**. On HAM10000, the optimal deep feature vector is tested. **Table 6** shows that Fine KNN performed well in this test, with an accuracy level of 99.3%. Compared to its competitors, it has a recall rate of 99.38%, a precision rate of 99.4%, an F1-Score of 99.38, and an FNR of 0.62%. Furthermore, the Fine KNN classifier’s compute time during the training phase is 939.93 seconds (sec), which is very good given its accuracy level. However, the Fine Tree classifier has the shortest computation time of 54.197 seconds. The outcome of this test demonstrates the importance of using BOA, as classification time is significantly reduced when comparing **Table 6** to **Tables 3**–**5**. **Figure 8** depicts a confusion matrix representing the Quadratic SVM’s recall rate. **Figure 8** depicts the accurately predicted values for each category in a diagonal format. The accuracy and time charts are shown in **Figures 9**, **10**, respectively. Both figures show that the feature fusion method in this work is critical for improving the accuracy level of all classifiers. In contrast, feature selection significantly reduces computation time with minor accuracy loss.

**Table 6:** Results of classification incorporating BOA feature selection on the HAM10000 dataset.

Classifiers	Accuracy (%)	Recall (%)	Precision (%)	F1 Score	FNR	Time (sec)
Narrow NN	98.8	98.95	98.95	98.95	1.05	140.77
Medium NN	98.9	99.02	99.04	99.02	0.98	121.21
Wide NN	99.0	99.1	99.11	99.10	0.9	175.26
Bilayered NN	98.8	98.97	98.95	98.95	1.02	230.11
Quadratic SVM	99.2	99.24	99.31	99.27	0.76	607.27
Cubic SVM	99.2	99.27	99.32	99.29	0.73	636.27
Coarse Gaussian SVM	98.8	98.94	99.01	98.97	1.06	997.09
**Fine KNN**	**99.3**	**99.38**	**99.4**	**99.38**	**0.62**	**939.93**
Ensemble Bagged Tree	98.7	98.87	98.88	98.87	1.13	211.26
Fine Tree	94.5	99.38	99.4	99.38	0.62	54.197

Bold show the significant value.

### 4.5 ISIC 2018 dataset results

The classification results on **ISIC 2018** using Xcpetion deep features are shown in **Table 7**. **Table 7** reflects that Cubic SVM performed well, attaining an accuracy level of 99.5%, while it also performs well on other metrics compared to its competitors, including the recall rate 82.58%, precision rate 88.6%, F1-Score 85.48, and last FNR 17.42%. Furthermore, the compute time of the Cubic SVM classifier during the training phase is 432.8 seconds (sec). However, the Fine Tree classifier has the least computation time, 31.110 seconds.

**Table 7:** Results of classification incorporating Xception deep features applied on the ISIC 2018 dataset.

Classifiers	Accuracy (%)	Recall (%)	Precision (%)	F1 Score	FNR	Time (sec)
Narrow NN	88.3	79.75	81.41	80.56	20.25	234.02
Medium NN	89.5	82.22	82.88	82.54	17.78	100.79
Wide NN	89.5	81.47	83.42	82.42	18.53	145.46
Bilayered NN	88.4	79.65	79.91	79.76	20.53	455.59
**Quadratic SVM**	**91.5**	**82.58**	**88.6**	**85.48**	**17.42**	**432.8**
Cubic SVM	91.5	82.5	88.51	85.38	17.5	490.1
Coarse Gaussian SVM	89.6	72.65	89.05	79.9	27.35	561.13
Fine KNN	87.8	81.71	79.27	80.46	18.29	175.58
Ensemble Bagged Tree	89.9	77.4	87.95	82.36	22.6	83.58
Fine Tree	83.4	64.55	71.44	67.82	35.45	31.110

Bold show the significant value.

The classification results on ISIC 2018 using Shufflent deep features are shown in **Table 8**. **Table 8** reflects that Cubic SVM achieves an accuracy level of 86.6%, whereas it also performs well on other metrics when compared with its competitors including the recall rate 71.71%, precision rate 80.91%, F1-Score 70.62, and FNR 28.29%. Furthermore, the compute time of the Cubic SVM classifier during the training phase is 163 seconds (sec). However, the Fine Tree classifier has the least computation time, which is 15.55 seconds.

**Table 8:** Results of classification incorporating Shufflenet deep features applied on the ISIC 2018 dataset.

Classifiers	Accuracy (%)	Recall (%)	Precision (%)	F1 Score	FNR	Time (sec)
Narrow NN	79.3	62.31	61.07	61.68	37.69	227.18
Medium NN	82.3	67.44	69.37	68.38	32.56	61.207
Wide NN	83.6	70.68	71.87	71.26	29.32	66
Bilayered NN	79.0	61.01	59.97	60.84	38.99	349.43
Quadratic SVM	86.4	71.91	80.52	75.96	28.09	145.91
**Cubic SVM**	**86.6**	**71.71**	**80.91**	**76.02**	**28.29**	**163**
Coarse Gaussian SVM	81.6	49.94	65.54	56.68	50.06	232.57
Fine KNN	81.7	71.07	68.78	69.9	28.93	66.811
Ensemble Bagged Tree	79.3	46.87	76.02	57.98	53.13	44.319
Fine Tree	72.6	39.98	49.34	44.16	60.02	15.55

Bold show the significant value.

The classification results on ISIC 2018 using combinatorial deep features extracted from Xception and ShuffleNet architectures are given in **Table 9**. In this test, **Table 9** reflects that Cubic SVM performed well with 91.9% accuracy, whereas it also performs well on other metrics when compared with its competitors including the recall rate 83.14%, precision rate 89.27%, F1-Score 86.08, and FNR 16.86. Furthermore, the compute time of the Cubic SVM classifier during the training phase is 1,051.4 seconds (sec). However, the Fine Tree classifier has the least computation time, which is 40.328 seconds.

**Table 9:** Results of classification incorporating feature fusion of both deep models on the ISIC 2018.

Classifiers	Accuracy (%)	Recall (%)	Precision (%)	F1 Score	FNR	Time (sec)
Narrow NN	89.2	81.6	83.87	82.7	18.84	263.41
Medium NN	90.2	83.22	84.25	83.72	16.78	129.93
Wide NN	90.4	84.54	84.72	84.62	15.46	158.44
Bilayered NN	89.2	80.51	80.91	80.7	19.49	447.38
Quadratic SVM	91.7	83.02	88.81	85.8	16.98	911.21
**Cubic SVM**	**91.9**	**83.14**	**89.27**	**86.08**	**16.86**	**1051.4**
Coarse Gaussian SVM	89.7	72.87	89.34	80.26	27.13	1228
Fine KNN	88.4	84.18	79.15	81.58	15.82	216.43
Ensemble Bagged Tree	89.7	77.17	87.6	82.04	22.83	98.32
Fine Tree	83.4	63.8	73.57	68.32	36.2	40.328

Bold show the significant value.

**Figure 11:**
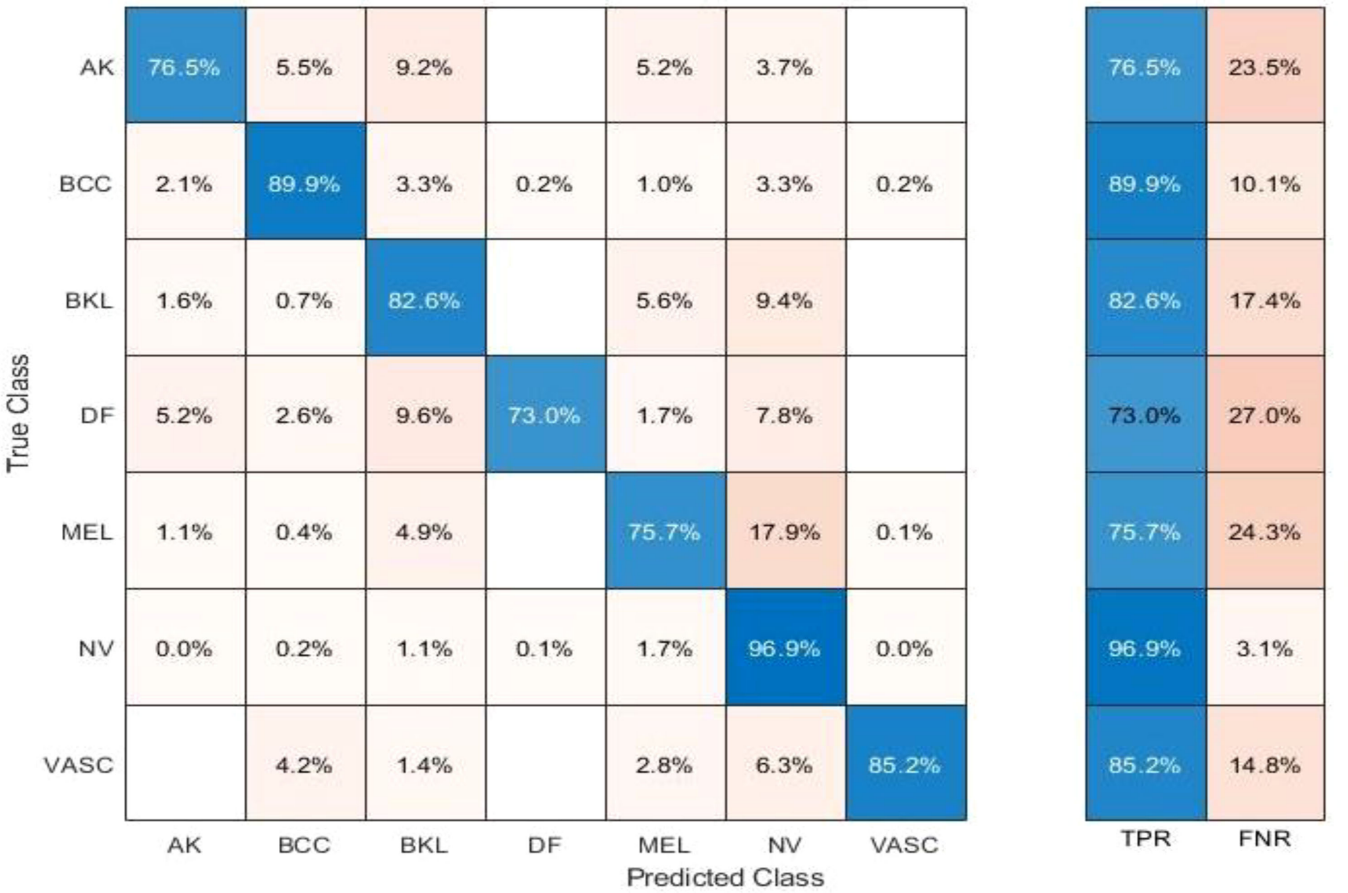
Confusion matrix of recall on Cubic SVM.

**Table 10** presents the results of feature selection using the optimization algorithm BOA. The resultant optimal deep feature vector is tested on the ISIC 2018 dataset. In this test, **Table 10** reflects that Cubic SVM accuracy is 91.5%, whereas it also performs well on other metrics when compared with its competitors including the recall rate 82.82%, precision rate 88.84%, F1-Score 88.82, and FNR 17.18. Furthermore, the compute time of the Cubic SVM classifier during the training phase is 239.72 seconds (sec). The Fine Tree classifier has the least computation time, which is 16.319 seconds. The result of this test clearly reflects the eminence of using BOA that classification time is considerably reduced when **Table 10** is compared with **Tables 7**–**9**, respectively. **Figure 11** shows a confusion matrix that represents the recall rate of the Cubic SVM. The accurate predicted values for each category are represented in diagonal in within **Figure 11**.

**Table 10:** Results of classification utilizing BOA feature selection on the ISIC 2018 dataset.

Classifiers	Accuracy (%)	Recall (%)	Precision (%)	F1 Score	FNR	Time (sec)
Narrow NN	88.0	79.97	79.52	79.74	20.03	121.25
Medium NN	88.9	81.68	81.6	81.63	18.32	54.922
Wide NN	89.5	82.77	82.58	82.67	17.23	70.606
Bilayered NN	87.9	79.35	80.07	79.70	20.65	214.52
Quadratic SVM	91.4	82.52	88.82	88.55	17.48	215.54
**Cubic SVM**	**91.5**	**82.82**	**88.84**	**88.82**	**17.18**	**239.72**
Coarse Gaussian SVM	89.6	71.62	89.51	79.57	28.38	273.64
Fine KNN	87.6	82.44	77.31	79.79	17.56	90.202
Ensemble Bagged Tree	89.4	75.85	86.91	81.00	24.15	48.395
Fine Tree	83.4	82.44	76.32	79.32	17.56	16.319

Bold show the significant value.

**Figure 12:**
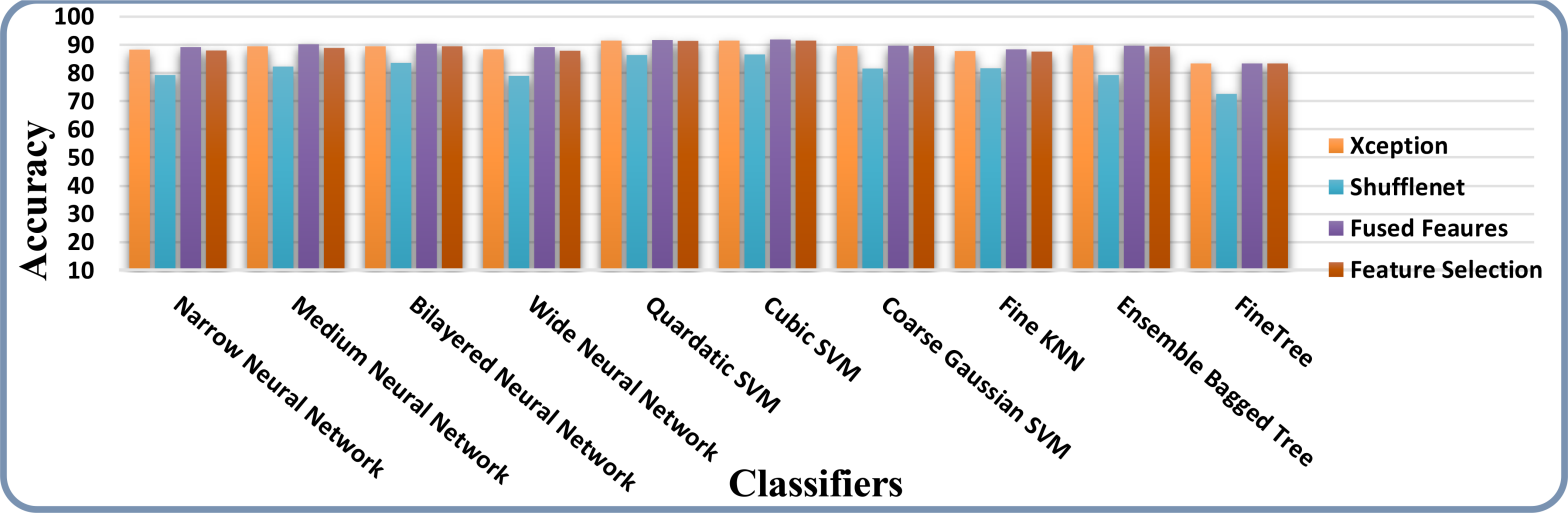
Accuracy comparison of all the intermediate steps on the ISIC 2018 dataset.

**Figure 13:**
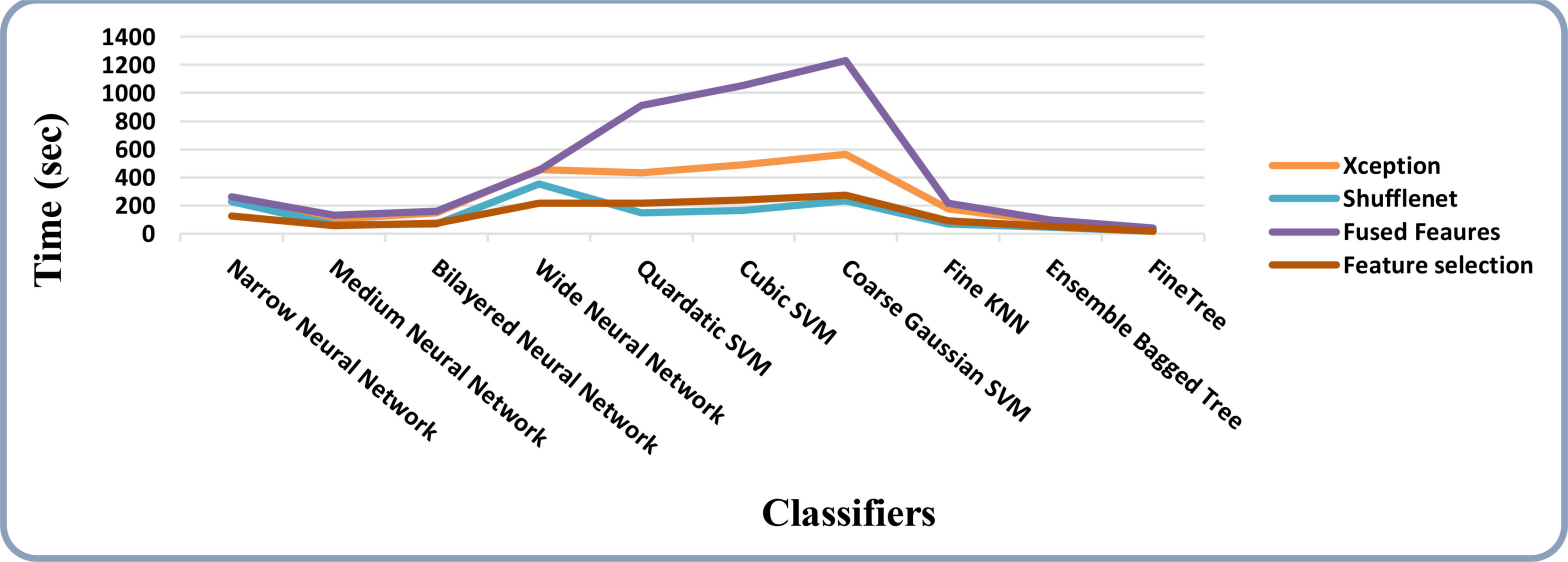
Time-based comparison of all the intermediate steps on the ISIC 2018 dataset.

The accuracy and time charts are illustrated in **Figures 12**, **13**, respectively. Both figures demonstrate that the feature fusion method in this work plays a vital role to improve the accuracy level of all the classifiers, whereas feature selection demonstrates a considerable reduction in computation time with minor accuracy loss.

### 4.6 Comparison with existing state-of-the-art proposed methods

**Table 11** shows a listing of the most recent techniques applied to a similar experimental dataset. Particularly in **Table 11**, Khan et al. (57) conducted their experiment using the HAM10000 dataset, reaching an accuracy of 96.5%. Bibi et al. (58) successfully achieved an accuracy of 96.7% for the HAM10000 dataset. Finally, Qureshi et al. (59) achieved a higher accuracy on the HAM10000 dataset, 92.83%. The researchers of (60–62) presented a deep learning–based system by utilizing the ISIC 2018 dataset, and they attained an accuracy of 86.2%, 90%, and 89.5%. In the approach that has been proposed, the accuracy that has been reached is 99.3% on the HAM10000 dataset and 91.5% on the ISIC2018 dataset. **Table 11** demonstrates that the proposed method achieves higher levels of accuracy than the techniques that have been used previously.

**Table 11:** Comparison of existing proposed methods with our proposed method.

Reference	Year	Datasets	Accuracy
(57)	2021	HAM10000	96.5%
(58)	2022	HAM10000	96.7%
(59)	2022	HAM10000	98.81%
**Proposed**		**HAM10000**	**99.3%**
(60)	2020	ISIC 2018	86.2%
(61)	2021	ISIC 2018	90%
(62)	2022	ISIC 2018	89.5%
**Proposed**		**ISIC 2018**	**91.5%**

Bold values represents the best accuracy.

**Figure 14:**
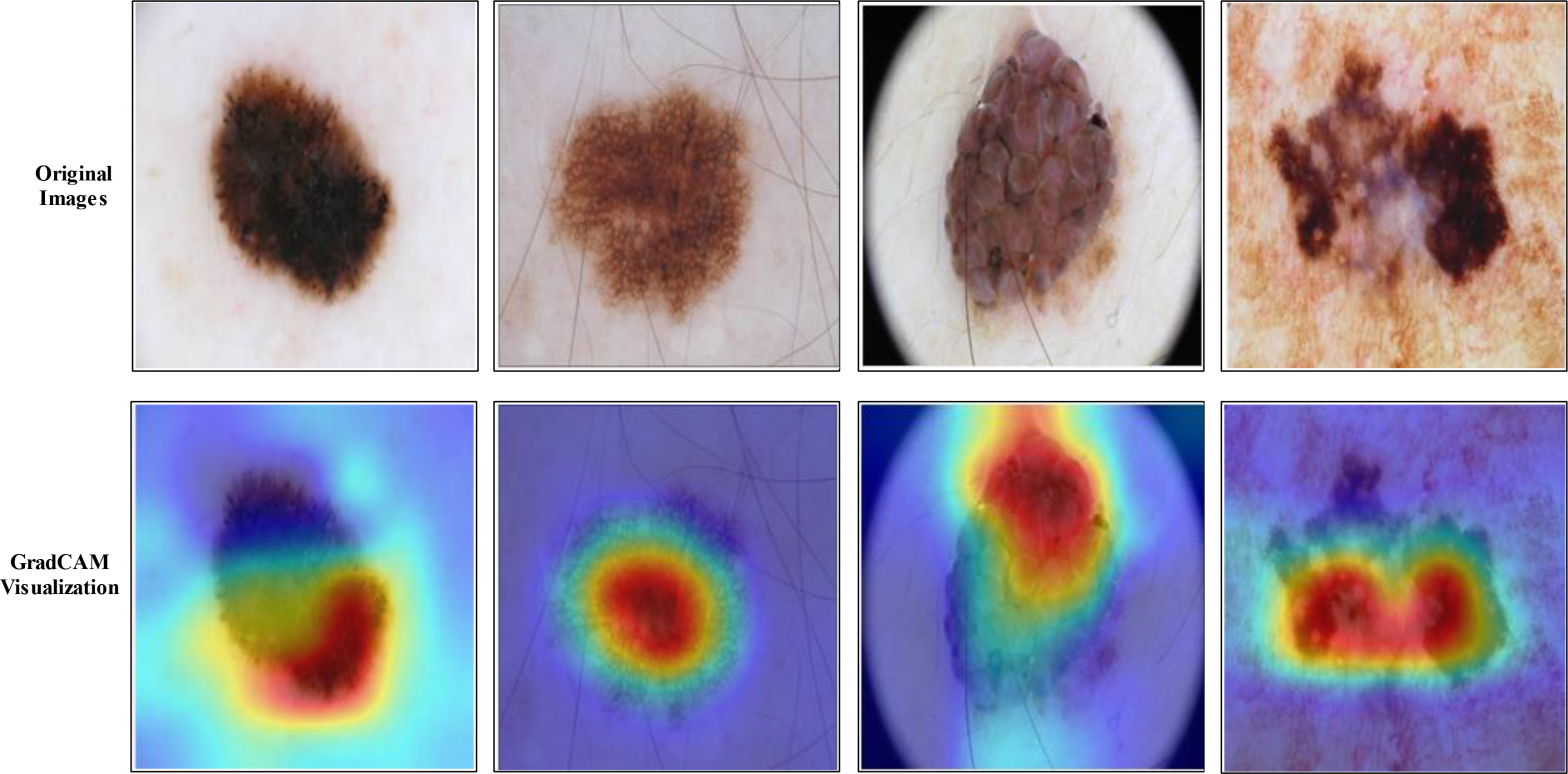
GradCAM-based visualization shows that the brown color is the most important part.

**Figure 15:**
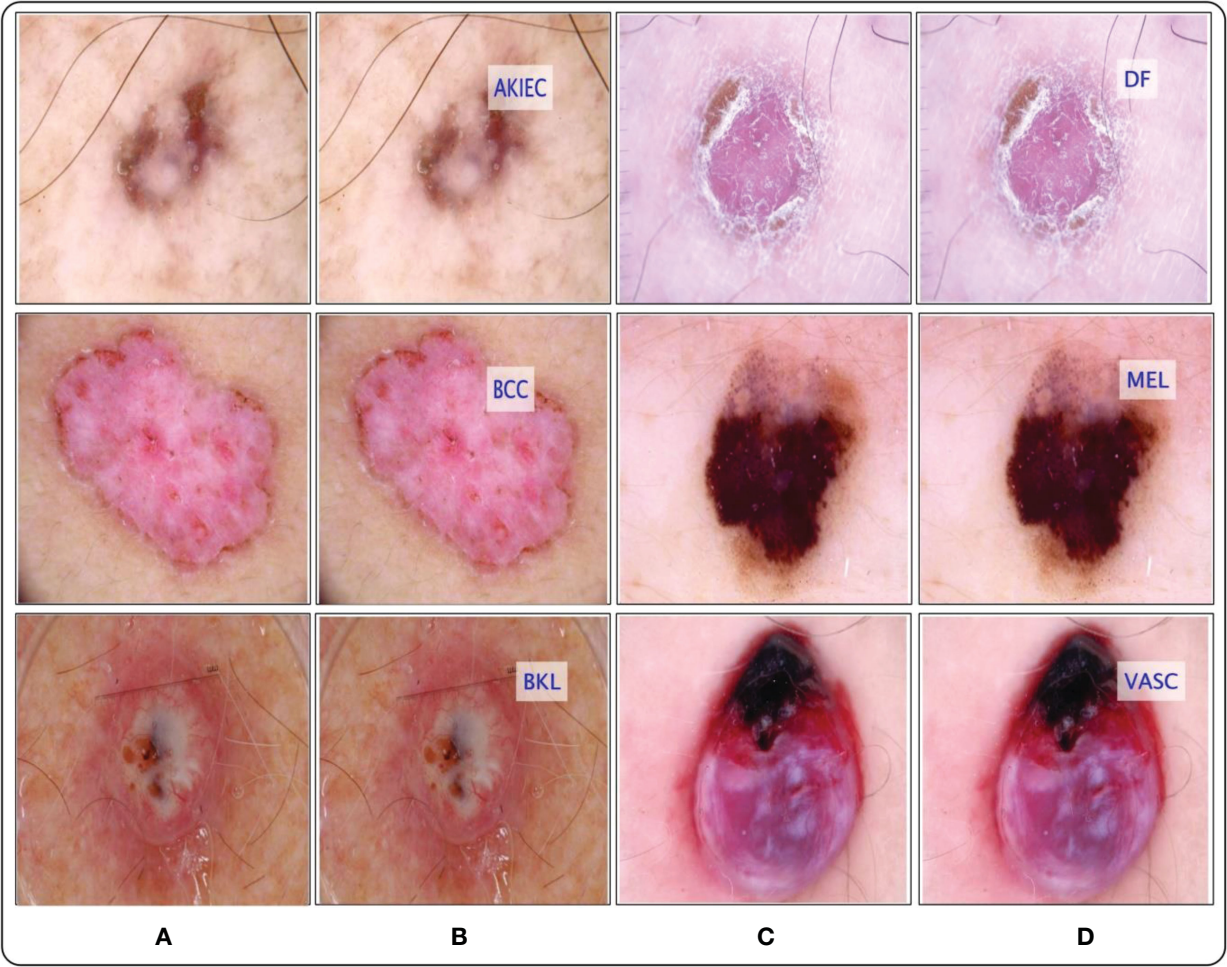
Proposed framework labeled results. **(A)** and **(C)** showing the original images, whereas the **(B)** and **(D)** show the proposed predicted labeled image.

The selection of the most optimal features is the main strength of this work. The BOA algorithm’s feature selection gives the best features with the best outcomes. Therefore, reducing high computational time and choosing the best optimal features are the primary focus of our research work. The most significant constraint of this study is a large amount of processing time required because of the increased number of features used in feature fusion. **Figure 14** shows the GradCAM based visualization. In this figure, the highlighted part with brown color shows the most important region. If this region features are not correctly extracted, then it is a chance that the classification error rate will be increased. Finally, the prediction results of proposed framework are shown in **Figure 15**.

## 5 Conclusions

Skin cancer has been one of the most crucial diseases in the world over the years, and it is considered a huge threat to human life. However, manual diagnosis methods to detect skin lesions are time-consuming, costly, and prone to errors due to the involvement of a dermatologist. A novel framework is proposed in this work for skin lesion classification using deep learning and explainable AI. Data augmentation was performed initially to improve the learning capability of deep learning models such as Xception and ShuffleNet. Then, features are extracted from the average pool layer of both deep models and fused to improve accuracy. The fusion step improved accuracy but, on the other hand, increased the computational time. Therefore, a feature selection algorithm is developed named improved BOA. The experiments were performed on two public datasets named HAM10000 and ISIC2018 and attained an accuracy of 99.3% and 91.5%, respectively. We compared recent techniques and showed that the proposed framework improved accuracy. In addition, GradCAM visualization shows that better results can be obtained if we initialize the hyperparameters of the deep models using an automated approach.

In the future, Bayesian optimization will be employed and parallel fusion techniques are proposed. Further, the proposed technique will be applied to public datasets, including ISIC 2019, PH2, and ISIC 2020.

## Data availability statement

The original contributions presented in the study are included in the article/supplementary material. Further inquiries can be directed to the corresponding authors.

## Author contributions

All authors listed have made a substantial, direct, and intellectual contribution to the work, and approved it for publication.
